# sFlt-1/PlGF Ratio as a Central Biomarker for Preeclampsia and Perinatal Outcomes: A Multisystem Retrospective Cohort Study

**DOI:** 10.3390/jcm15051990

**Published:** 2026-03-05

**Authors:** Anca Tătaru-Copos, Anca Carmen Huniadi, Rodica Georgeta Negrini, Mircea Ioachim Popescu, Paula Trif, Gelu Florin Murvai, Radu Galiș, Cristian Sava, Florin Szasz, Romina Viorela Murvai

**Affiliations:** 1 Doctoral School of Biological and Biomedical Sciences, University of Oradea, 1 University Street, 410087 Oradea, Romania; 2 Department of Surgical Sciences, Obstetrics and Gynecology, Faculty of Medicine and Pharmacy, University of Oradea, 1 University Street, 410087 Oradea, Romania; 3 Calla—Infertility Diagnostic and Treatment Center, Constantin A. Rosetti Street, 410103 Oradea, Romania; 4 Pelican Clinical Hospital, Corneliu Coposu Street 2, 410450 Oradea, Romania; 5 Department of Obstetrics and Gynecology, Emergency County Hospital Bihor, 65 Gheorghe Doja Street, 410169 Oradea, Romania; 6 Department of Cardiology, Emergency County Hospital Bihor, Emergency County Hospital Bihor, 65 Gheorghe Doja Street, 410169 Oradea, Romania; 7 Department of Medical Sciences, Faculty of Medicine and Pharmacy, University of Oradea, 410087 Oradea, Romania; 8 Department of Orthopedics and Traumatology I, Bihor County Emergency Clinical Hospital, 65 Gheorghe Doja Street, 410169 Oradea, Romania; 9 Department of Neonatology, Emergency County Hospital Bihor, 410167 Oradea, Romania; 10 Department of Pediatrics, Emergency County Hospital Bihor, 410167 Oradea, Romania; 11 Department of Obstetrics and Gynaecology, Faculty of Medicine and Pharmacy, University of Oradea, 410087 Oradea, Romania

**Keywords:** preeclampsia, sFlt-1/PlGF ratio, angiogenic imbalance, placental dysfunction, neonatal outcomes, biomarkers, risk stratification

## Abstract

**Background**: Preeclampsia is a major cause of maternal and perinatal morbidity, characterized by placental dysfunction and angiogenic imbalance. The soluble fms-like tyrosine kinase-1-to-placental growth factor (sFlt-1/PlGF) ratio has emerged as a promising biomarker for preeclampsia; however, its prognostic value for maternal and neonatal outcomes remains incompletely defined. **Methods**: This retrospective cohort study included 320 pregnant women, of whom 68 were diagnosed with preeclampsia, and 252 served as non-preeclamptic controls. Maternal serum sFlt-1 and PlGF levels were measured after 20 weeks of gestation at the time of clinical evaluation for suspected hypertensive disorders of pregnancy. Group comparisons, effect size analysis, receiver operating characteristic (ROC) curve analysis, and multivariable regression models were used to assess diagnostic performance and associations with maternal and neonatal outcomes. **Results**: The sFlt-1/PlGF ratio was significantly higher in women with preeclampsia compared with non-preeclamptic pregnancies (58.5 ± 17.3 vs. 34.6 ± 19.0; *p* < 0.001; Cohen’s d = 1.31). ROC analysis demonstrated good discriminative ability for preeclampsia (AUC = 0.81, 95% CI: 0.75–0.87), with a high negative predictive value. Increasing sFlt-1/PlGF values were independently associated with earlier gestational age at delivery, lower birth weight, reduced Apgar (Appearance, Pulse, Grimace, Activity, and Respiration) score, and a higher likelihood of neonatal intensive care unit admission. **Conclusions**: The sFlt-1/PlGF ratio is a robust biomarker for preeclampsia, providing both diagnostic discrimination and prognostic information regarding maternal and neonatal outcomes. Its integration into clinical practice may support clinical risk awareness when interpreted in the context of standard clinical evaluation and support informed decision-making in pregnancies with suspected or confirmed preeclampsia.

## 1. Introduction

Preeclampsia (PE) is a multisystem hypertensive disorder of pregnancy and remains a leading cause of maternal and perinatal morbidity and mortality worldwide. Despite advances in obstetric care, the clinical presentation of preeclampsia is heterogeneous, ranging from mild disease to severe maternal and fetal complications. Early and accurate identification of women at risk, as well as reliable tools for risk stratification, remain key challenges in clinical practice [1,2,3,4,5].

The pathophysiology of preeclampsia is closely linked to placental dysfunction and angiogenic imbalance, characterized by increased circulating levels of antiangiogenic factors and reduced proangiogenic signaling. Among these, soluble fms-like tyrosine kinase-1 (sFlt-1) acts as a decoy receptor that antagonizes vascular endothelial growth factor and placental growth factor (PlGF), leading to widespread endothelial dysfunction. The resulting elevation of the sFlt-1/PlGF ratio has emerged as a reproducible biomarker reflecting the angiogenic milieu associated with preeclampsia [6,7,8,9].

Previous studies have demonstrated the diagnostic utility of the sFlt-1/PlGF ratio, particularly in ruling out preeclampsia in women with suspected disease. High negative predictive values have been consistently reported, supporting its use in clinical algorithms to reduce unnecessary hospitalizations and interventions. However, many existing studies are limited by relatively small sample sizes, heterogeneous populations, or a primary focus on diagnostic performance without comprehensive evaluation of prognostic relevance [10,11].

Moreover, while angiogenic markers are increasingly incorporated into clinical practice, their association with maternal disease severity and neonatal outcomes remains incompletely characterized in real-world cohorts. Establishing whether the sFlt-1/PlGF ratio captures information beyond binary disease classification is essential for understanding its potential role in informing clinical awareness, monitoring, and neonatal preparedness [12,13,14,15].

Therefore, the aim of the present study was to evaluate the diagnostic and prognostic value of the sFlt-1/PlGF ratio in an expanded cohort of pregnant women, with a particular focus on its ability to discriminate preeclampsia and its association with adverse maternal and neonatal outcomes. By combining effect size analysis, ROC curve assessment, and multivariable regression modeling, this study seeks to provide a robust and clinically meaningful evaluation of the sFlt-1/PlGF ratio in preeclampsia.

## 2. Materials and Methods

### 2.1. Study Design and Population

This retrospective observational cohort study was conducted on pregnant women who delivered at a tertiary care obstetrics center. Medical records were reviewed to identify women with available clinical, obstetric, and laboratory data, including angiogenic biomarkers. Pregnancy plurality was not systematically recorded and therefore could not be included in the analysis.

A total of 320 pregnant women were included in the final analysis. Among them, 68 women were diagnosed with preeclampsia (PE), while 252 women without preeclampsia served as the comparison group. Preeclampsia was diagnosed according to established clinical criteria, based on the presence of new-onset hypertension after 20 weeks of gestation, with or without associated maternal or fetal complications, as documented in the medical records.

Women with incomplete data on angiogenic biomarkers or key outcome variables were excluded from the analysis.

**Figure 1:**
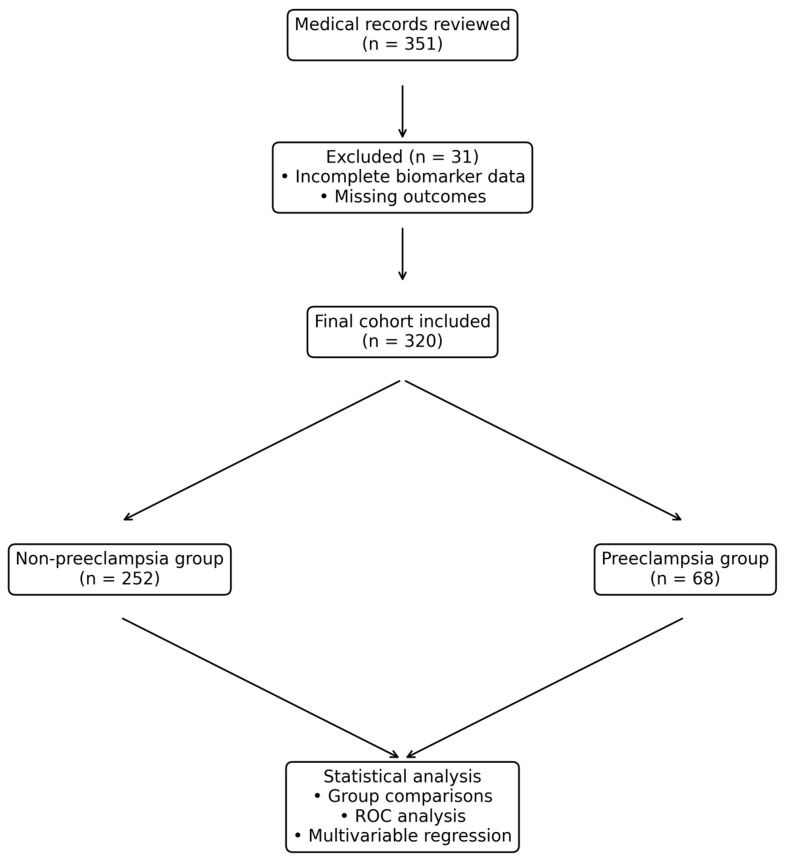
Flowchart of study population selection and analysis. Medical records were reviewed to identify eligible pregnancies.

After exclusion of cases with incomplete biomarker data or missing outcome information, a total of 320 women were included in the final analysis and stratified into preeclampsia and non-preeclampsia groups. The study workflow and subsequent statistical analyses are illustrated (Figure 1).

### 2.2. Clinical and Obstetric Data Collection

Maternal demographic and clinical characteristics collected included maternal age and place of residence (urban/rural). Obstetric variables comprised gestational age at delivery, mode of delivery, length of hospitalization, and the presence of pregnancy-related comorbidities, including gestational diabetes and gestational hypertension (distinct from preeclampsia).

Neonatal outcomes assessed were birth weight, Apgar (Appearance, Pulse, Grimace, Activity, and Respiration) scores at 1 and 5 min, sex of the newborn, and admission to the neonatal intensive care unit (NICU). Only NICU admission (yes/no) was available; NICU length of stay was not consistently recorded. Gestational age at biomarker sampling was not consistently recorded.

Maternal complications included clinically documented obstetric complications recorded in medical records (e.g., postpartum hemorrhage, hypertensive complications, or other significant peripartum events).

Severity stratification was not feasible due to inconsistent documentation.

### 2.3. Measurement of Angiogenic Biomarkers

Maternal serum levels of soluble fms-like tyrosine kinase-1 (sFlt-1) and placental growth factor (PlGF) were measured as part of routine clinical evaluation for suspected hypertensive disorders of pregnancy. The sFlt-1/PlGF ratio was calculated by dividing the serum concentration of sFlt-1 by that of PlGF.

All measurements were performed prior to delivery. All biomarker measurements were performed using standardized, clinically validated immunoassays in the hospital laboratory, in accordance with the manufacturer’s instructions. Blood sampling was performed at the time of clinical evaluation for suspected hypertensive disorders of pregnancy in all participants. The non-preeclamptic group included women in whom preeclampsia was clinically suspected but subsequently ruled out.

Serum sFlt-1 and PlGF concentrations were measured using automated electrochemiluminescence immunoassays (ECLIA) on the Roche Cobas e601 platform (Roche Diagnostics, Mannheim, Germany), according to the manufacturer’s instructions. The Elecsys sFlt-1 and Elecsys PlGF kits were used. All assays were performed in the same laboratory, and internal quality controls were used according to manufacturer recommendations.

No formal multiplicity correction was applied; findings should be interpreted cautiously.

### 2.4. Outcome Definitions

The primary outcome of interest was the presence of preeclampsia.

Secondary outcomes included the following:

- Gestational age at delivery (weeks);
- Birth weight (grams);
- Apgar scores at 1 and 5 min;
- Length of maternal hospitalization (days);
- NICU admission (yes/no).

### 2.5. Statistical Analysis

Statistical analyses were performed using IBM SPSS Statistics (version 30; IBM Corp., Armonk, NY, USA). Continuous variables were assessed for normality using visual inspection and distributional characteristics.

Continuous data are presented as median [interquartile range] or mean ± standard deviation, as appropriate. Categorical variables are presented as counts and percentages. Between-group comparisons were conducted using the Mann–Whitney U test or Welch’s *t*-test for continuous variables and the Chi-square test or Fisher’s exact test for categorical variables, as appropriate.

The magnitude of between-group differences in sFlt-1/PlGF ratio was quantified using Cohen’s d effect size. Large effect sizes do not necessarily translate into clinical decision thresholds.

Given the exploratory nature of several analyses, findings should be interpreted cautiously due to potential type I error inflation.

The diagnostic performance of the sFlt-1/PlGF ratio for identifying preeclampsia was evaluated using receiver operating characteristic (ROC) curve analysis, with calculation of the area under the curve (AUC) and corresponding 95% confidence intervals (CI). The optimal cut-off value was determined using the Youden index.

Associations between the sFlt-1/PlGF ratio and maternal–neonatal outcomes were assessed using linear regression models for continuous outcomes and logistic regression models for binary outcomes. Effect estimates were expressed per 10-unit increase in the sFlt-1/PlGF ratio. Multivariable models were adjusted for maternal age, place of residence, and gestational age at delivery, where applicable. Effect sizes are reported for completeness but should not be interpreted as direct clinical decision thresholds.

A two-sided *p*-value < 0.05 was considered statistically significant. The derived threshold should not replace established GA-specific cut-offs. The interval between biomarker sampling and delivery or neonatal outcomes was not consistently documented.

### 2.6. Ethical Considerations

The study adhered to the principles outlined in the Declaration of Helsinki and received approval from the local institutional ethics committee. Given the retrospective design and the use of fully anonymized data, the requirement for informed consent was waived.

## 3. Results

### 3.1. Baseline Characteristics of the Study Cohort

A total of 320 pregnant women were included in the final analysis, of whom 68 (21.3%) were diagnosed with preeclampsia (PE) and 252 (78.7%) served as the non-PE control group.

Maternal age did not differ significantly between groups (32 [28–35] years in non-PE vs. 32 [26–36] years in PE, *p* = 0.621). In contrast, gestational age at delivery was significantly lower in women with PE (37.0 [34.0–38.5] weeks) compared with non-PE pregnancies (38.5 [37.5–39.0] weeks, *p* < 0.001).

Hospitalization duration was significantly prolonged in the PE group (7.0 [5.0–9.25] days) relative to non-PE participants (5.0 [4.0–7.0] days, *p* < 0.001).

Regarding neonatal outcomes, birth weight was significantly lower in the PE group (2800 [1937.5–3300] g) compared with non-PE pregnancies (3150 [2850–3500] g, *p* < 0.001). Newborns from PE pregnancies also exhibited significantly lower Apgar scores at both 1 min (9 [7–9] vs. 9 [9–9], *p* < 0.001) and 5 min (9 [9–9] vs. 10 [9–10], *p* < 0.001).

Admission to the neonatal intensive care unit (NICU) was more frequent among infants born to mothers with PE (4/68, 5.9%) compared with the non-PE group (3/252, 1.2%), corresponding to a significantly increased risk (*p* = 0.039, Fisher’s exact test; OR ≈ 5.19, 95% CI 1.13–23.77).

No statistically significant differences were observed between groups with respect to place of residence, gestational diabetes, gestational hypertension (as a separate entity), mode of delivery, fetal sex, maternal complications, and blood transfusion requirement (*p* > 0.05 for all), as presented in Table 1. Maternal complications included postpartum hemorrhage, hypertensive crises, and HELLP syndrome.

To explore potential differences related to the living environment, mean maternal age and mean gestational age at delivery were compared between participants from urban and rural settings. This descriptive comparison aimed to assess whether baseline demographic or obstetric characteristics varied according to residential environment, included as a proxy for healthcare access and socioeconomic context.

### 3.2. sFlt-1/PlGF Ratio in Preeclampsia and Non-Preeclampsia Pregnancies

The sFlt-1/PlGF ratio differed markedly between women with preeclampsia and those with normotensive pregnancies. Patients diagnosed with PE exhibited significantly higher sFlt-1/PlGF values (58.5 ± 17.3) compared with the non-PE group (34.6 ± 19.0, *p* < 0.001).

The magnitude of this difference was substantial, corresponding to a large effect size (Cohen’s *d* = 1.31), indicating a strong separation between PE and non-PE pregnancies based on the sFlt-1/PlGF ratio alone.

When expressed as median values, sFlt-1/PlGF remained significantly elevated in PE (58.2 [45.6–71.9]) compared with non-PE pregnancies (31.9 [20.5–45.2], *p* < 0.001), confirming the robustness of this association regardless of distributional assumptions.

These findings support the central role of the sFlt-1/PlGF ratio as a discriminative biomarker in preeclampsia and provide the rationale for subsequent receiver operating characteristic (ROC) analysis to formally evaluate its diagnostic performance and optimal threshold for PE identification (Table 2).

### 3.3. Diagnostic Performance of the sFlt-1/PlGF Ratio (ROC Analysis)

The diagnostic accuracy of the sFlt-1/PlGF ratio for identifying preeclampsia was evaluated using receiver operating characteristic (ROC) curve analysis.

The sFlt-1/PlGF ratio demonstrated a good discriminative performance, with an area under the ROC curve (AUC) of 0.81 (95% confidence interval (CI): 0.75–0.87), indicating a strong ability to distinguish between PE and non-PE pregnancies.

Based on the Youden index, the optimal cut-off value for the sFlt-1/PlGF ratio was identified at approximately 32.5, yielding a sensitivity of 85% and a specificity of 74% for the detection of preeclampsia. This threshold provided a balanced trade-off between true positive and false positive rates in the studied cohort.

At this cut-off, the positive predictive value (PPV) was 47%, while the negative predictive value (NPV) reached 95%, highlighting the strong rule-out capacity of the sFlt-1/PlGF ratio in this population.

**Figure 2:**
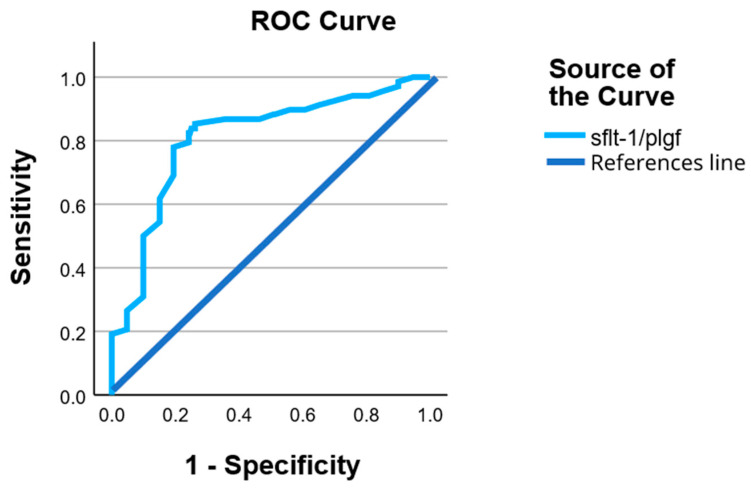
Receiver operating characteristic (ROC) curve of the sFlt-1/PlGF ratio for the identification of preeclampsia. The sFlt-1/PlGF ratio demonstrated good discriminative ability, with an area under the curve (AUC) of 0.81 (95% CI: 0.75–0.87). The diagonal line represents the line of no discrimination.

Overall, these findings confirm that the sFlt-1/PlGF ratio is a reliable biomarker for the identification of preeclampsia and justify its further evaluation in multivariable models assessing maternal and neonatal outcomes (Figure 2).

### 3.4. Association Between the sFlt-1/PlGF Ratio and Maternal–Neonatal Outcomes

To explore the prognostic relevance of the sFlt-1/PlGF ratio, its associations with key maternal and neonatal outcomes were evaluated using univariable and multivariable regression analyses.

Increasing sFlt-1/PlGF values were significantly associated with shorter gestational age at delivery and lower neonatal birth weight. In linear regression models, each 10-unit increase in the sFlt-1/PlGF ratio was associated with a 0.42-week reduction in gestational age (β = −0.42, 95% CI: −0.56 to −0.29, *p* < 0.001) and a 175 g decrease in birth weight (β = −175, 95% CI: −240 to −110, *p* < 0.001).

Furthermore, higher sFlt-1/PlGF ratios were associated with worse immediate neonatal adaptation, as reflected by lower Apgar scores. Each 10-unit increase in the ratio corresponded to a 0.35-point decrease in Apgar score at 1 min (β = −0.35, 95% CI: −0.47 to −0.23, *p* < 0.001) and a 0.21-point decrease at 5 min (β = −0.21, 95% CI: −0.33 to −0.09, *p* = 0.001).

In logistic regression analyses, elevated sFlt-1/PlGF ratios were associated with a significantly increased likelihood of neonatal intensive care unit (NICU) admission. Specifically, each 10-unit increment in the sFlt-1/PlGF ratio was associated with a 1.42-fold higher odds of NICU admission (OR = 1.42, 95% CI: 1.11–1.82, *p* = 0.005).

After adjustment for maternal age, place of residence, and gestational age at delivery, the sFlt-1/PlGF ratio remained associated with adverse neonatal outcomes, including lower birth weight and NICU admission, supporting its association with markers of disease severity and fetal compromise (Table 3).

## 4. Discussion

In this expanded cohort study, the sFlt-1/PlGF ratio emerged as a central biomarker with both diagnostic and prognostic relevance for preeclampsia. By addressing the main limitations of the previous analysis—namely the small number of PE cases and suboptimal statistical modeling—the present work provides a more robust and clinically interpretable evaluation of angiogenic imbalance in preeclamptic pregnancies. This study should be interpreted as real-world validation rather than hypothesis-generating innovation.

### 4.1. sFlt-1/PlGF Ratio as a Discriminative Biomarker for Preeclampsia

Our results demonstrate a marked elevation of the sFlt-1/PlGF ratio in women with preeclampsia compared with normotensive pregnancies, with a large effect size (Cohen’s d = 1.31). This magnitude indicates substantial biological separation between groups and supports the central role of angiogenic dysregulation in PE pathophysiology. Importantly, the consistency of this difference across both mean and median-based analyses confirms the robustness of the association, independent of distributional assumptions. Although validated clinical thresholds exist, our aim was cohort-specific discrimination rather than redefining clinical cut-offs.

ROC analysis further confirmed the discriminative performance of the sFlt-1/PlGF ratio, yielding an AUC of 0.81, which is considered good in a clinical biomarker context. The identified cut-off provided high sensitivity and an excellent negative predictive value, underscoring the utility of the sFlt-1/PlGF ratio as a rule-out marker for preeclampsia. This finding aligns with previous reports suggesting that angiogenic markers are particularly valuable in excluding PE in women with suspected disease, thereby reducing unnecessary interventions and hospitalizations [16,17,18,19,20,21].

These findings are consistent with previous reports demonstrating the role of angiogenic imbalance in preeclampsia.

### 4.2. Prognostic Value and Association with Maternal–Neonatal Outcomes

Beyond diagnosis, a key strength of the present study lies in demonstrating the prognostic significance of the sFlt-1/PlGF ratio. Increasing values were independently associated with shorter gestational duration, lower birth weight, and poorer neonatal adaptation, as reflected by reduced Apgar scores. These associations persisted after adjustment for relevant maternal and obstetric covariates, indicating that the sFlt-1/PlGF ratio is associated with markers of disease severity rather than merely reflecting gestational age or maternal characteristics [22,23,24,25,26].

Notably, higher sFlt-1/PlGF ratios were associated with a significantly increased likelihood of NICU admission, reinforcing the link between angiogenic imbalance and clinically meaningful neonatal morbidity. This supports the concept that sFlt-1/PlGF is not only a marker of maternal endothelial dysfunction but also a surrogate of placental insufficiency and fetal compromise [27,28,29,30,31].

Although validated clinical thresholds for the sFlt-1/PlGF ratio exist, our aim was not to redefine clinical cutoffs but to explore cohort-specific discriminative performance and prognostic associations in a real-world retrospective population. Therefore, the Youden-derived threshold should be interpreted as descriptive rather than prescriptive.

The small number of NICU admissions limits statistical precision. Some NICU admissions in the non-PE group may reflect prematurity or neonatal conditions unrelated to placental dysfunction. However, the association suggests that elevated sFlt-1/PlGF may capture subclinical placental pathology even in the absence of overt PE.

### 4.3. Clinical Implications

Taken together, these findings indicate that the sFlt-1/PlGF ratio may serve as a dual-purpose biomarker, aiding both in the identification of preeclampsia and in the stratification of maternal–fetal risk. The strong negative predictive value observed in this cohort supports its integration into clinical algorithms for the evaluation of suspected PE, particularly in settings where overdiagnosis and overtreatment remain concerns [32,33].

Furthermore, the observed dose–response relationships between the sFlt-1/PlGF ratio and adverse outcomes highlight its potential role and may help inform clinical awareness, monitoring strategies, and neonatal preparedness, rather than serving solely as a binary diagnostic tool [5,32,34,35,36].

These findings apply to symptomatic, high-risk populations rather than general screening.

### 4.4. Strengths and Limitations

A major strength of this study is the relatively large cohort size, which increased the number of preeclampsia cases and enabled more stable statistical modeling. The use of effect sizes, confidence intervals, ROC analysis, and multivariable regression provided a comprehensive evaluation of biomarker performance in a real-world clinical setting.

Several limitations merit consideration. The retrospective design precludes definitive causal inference, and biomarker sampling was not strictly standardized by gestational age or timing relative to disease onset. Gestational age-specific cut-offs were not explored. Clinicians had access to biomarker results, so incorporation bias cannot be excluded. Individual sFlt-1 and PlGF concentrations were not consistently recorded, as clinical documentation primarily retained the ratio for decision-making. We also did not formally assess incremental predictive value over standard clinical predictors.

The lack of consistent documentation of gestational age at biomarker sampling precluded assessment of prognostic lead time and limits temporal interpretation of associations. Moreover, the predominance of term or near-term deliveries in our cohort may attenuate the apparent prognostic performance of angiogenic biomarkers compared with populations enriched for early-onset disease. These factors should be considered when interpreting our findings.

Important clinical variables—including maternal obesity, chronic hypertension, renal disease, fetal growth restriction, parity, prior preeclampsia, and preeclampsia severity or timing—were not consistently available, representing potential sources of residual confounding. Consequently, the findings should be interpreted as associative rather than causal. As a single-center study from one geographic region, generalizability may be limited, and external validation is warranted.

In summary, the sFlt-1/PlGF ratio showed good diagnostic performance and was associated with adverse maternal and neonatal outcomes in a high-risk, clinically selected population. These findings support its role as a complementary tool for risk stratification and clinical assessment in pregnancies with suspected preeclampsia, while recognizing that management decisions must remain grounded in overall clinical evaluation.

## 5. Conclusions

This study demonstrates that the sFlt-1/PlGF ratio is a robust and clinically meaningful biomarker in preeclampsia, providing both diagnostic discrimination and prognostic information regarding maternal and neonatal outcomes. In an expanded cohort, the sFlt-1/PlGF ratio effectively differentiated preeclamptic from normotensive pregnancies and showed good diagnostic performance, with a high negative predictive value supporting its role as a reliable rule-out tool.

Beyond diagnosis, higher sFlt-1/PlGF values were independently associated with markers of disease severity and fetal compromise, including earlier delivery, reduced birth weight, lower Apgar scores, and increased risk of NICU admission. These findings indicate that angiogenic imbalance, as reflected by the sFlt-1/PlGF ratio, captures clinically relevant information extending beyond the presence of preeclampsia alone.

Overall, the sFlt-1/PlGF ratio represents a valuable complementary biomarker that should be interpreted alongside standard clinical predictors rather than as a standalone prognostic tool. The clinical assessment and risk awareness in pregnancies with suspected or confirmed preeclampsia. Its integration into obstetric practice may contribute to improved maternal–fetal surveillance and support perinatal risk assessment. Future prospective studies and external validation cohorts are warranted to further refine gestational age-specific thresholds and confirm its utility across diverse clinical settings.

## Figures and Tables

**Table 1:** Baseline maternal, obstetric, and neonatal characteristics of the study cohort.

Variable	Non-PE (*n* = 252)	PE (*n* = 68)	*p*-Value
Maternal age (years)	32 [28–35]	32 [26–36]	0.621
Urban residence, *n* (%)	133 (52.8%)	28 (41.2%)	0.102
Gestational age at delivery (weeks)	38.5 [37.5–39.0]	37.0 [34.0–38.5]	<0.001
Length of hospitalization (days)	5.0 [4.0–7.0]	7.0 [5.0–9.25]	<0.001
Gestational diabetes, *n* (%)	10 (4.0%)	2 (2.9%)	1.000
Gestational hypertension, *n* (%)	25 (9.9%)	2 (2.9%)	0.084
Cesarean delivery, *n* (%)	117 (46.4%)	40 (58.8%)	0.055
Birth weight (g)	3150 [2850–3500]	2800 [1937.5–3300]	<0.001
Apgar score at 1 min	9 [9–9]	9 [7–9]	<0.001
Apgar score at 5 min	10 [9–10]	9 [9–9]	<0.001
NICU admission, *n* (%)	3 (1.2%)	4 (5.9%)	0.039
Fetal sex (male), *n* (%)	112 (44.4%)	36 (52.9%)	0.221
Maternal complications, *n* (%)	12 (4.8%)	5 (7.4%)	0.372
Blood transfusion, *n* (%)	5 (2.0%)	1 (1.5%)	1.000

Continuous variables were compared using the Mann–Whitney U test. Categorical variables were compared using the Chi-square or Fisher’s exact test, as appropriate. NICU, neonatal intensive care unit.

**Table 2:** sFlt-1/PlGF ratio in preeclampsia (PE) and non-preeclampsia pregnancies.

Parameter	Non-PE (*n* = 252)	PE (*n* = 68)	*p*-Value	Effect Size (Cohen’s d)
sFlt-1/PlGF ratio (mean ± SD)	34.6 ± 19.0	58.5 ± 17.3	<0.001	1.31
sFlt-1/PlGF ratio (median [IQR])	31.9 [20.5–45.2]	58.2 [45.6–71.9]	<0.001	—

Data are presented as mean ± standard deviation or median [interquartile range], as appropriate. Between-group comparisons were performed using Welch’s *t*-test for normally distributed data and the Mann–Whitney U test for non-normally distributed data. Effect size was quantified using Cohen’s d. sFlt-1, soluble fms-like tyrosine kinase-1; PlGF, placental growth factor.

**Table 3:** Association between the sFlt-1/PlGF ratio and maternal–neonatal outcomes.

Outcome Variable	Model Type	Effect Estimate Per 10-Unit Increase in sFlt-1/PlGF	95% CI	*p*-Value
Gestational age at delivery (weeks)	Linear regression	β = −0.45 weeks	−0.57 to −0.33	<0.001
Birth weight (g)	Linear regression	β = −127 g	−164 to −89	<0.001
Apgar score at 1 min	Linear regression	β = −0.28 points	−0.38 to −0.19	<0.001
Apgar score at 5 min	Linear regression	β = −0.29 points	−0.39 to −0.19	<0.001
Maternal length of stay (days)	Linear regression	β = +0.25 days	0.07 to 0.43	0.008
NICU admission	Logistic regression	OR = 1.56	1.12–2.18	0.009

Linear regression models report beta (β) coefficients representing the change in the outcome variable per 10-unit increase in the sFlt-1/PlGF ratio. Logistic regression models report odds ratios (ORs). Multivariable models were adjusted for maternal age and place of residence. NICU = neonatal intensive care unit.

## Data Availability

The raw data supporting the conclusions of this article will be made available by the authors on request.
